# Classification and Clustering of Fiber Break Events in Thermoset CFRP Using Acoustic Emission and Machine Learning

**DOI:** 10.3390/s25206466

**Published:** 2025-10-19

**Authors:** Richard Dela Amevorku, David Amoateng-Mensah, Manoj Rijal, Mannur J. Sundaresan

**Affiliations:** Department of Mechanical Engineering, North Carolina A & T State University, 1601 E. Market Street, Greensboro, NC 27411, USA; rdamevorku@aggies.ncat.edu (R.D.A.); damoatengmensah@aggies.ncat.edu (D.A.-M.); mannur@ncat.edu (M.J.S.)

**Keywords:** acoustic emission (AE), damage mechanisms in thermoset composites, quasi-isotropic laminate, machine learning (ML), cross correlation, fiber break

## Abstract

Carbon Fiber-Reinforced Polymer (CFRP) composites, widely used across industries, exhibit various damage mechanisms depending on the loading conditions applied. This study employs a structural health monitoring (SHM) approach to investigate the three primary failure modes, fiber breakage, matrix cracking, and delamination, in thermoset quasi-isotropic CFRPs subjected to quasi-static tensile loading until failure. Acoustic emission (AE) signals acquired from an experiment were leveraged to analyze and classify these real-time signals into the failure modes using machine learning (ML) techniques. Due to the extensive number of AE signals recorded during testing, manually classifying these failure mechanisms through waveform inspection was impractical. ML, alongside ensemble learning, algorithms were implemented to streamline the classification, making it more efficient, accurate, and reliable. Conventional AE parameters from the data acquisition system and feature extraction techniques applied to the recorded waveforms were implemented exclusively as classification features to investigate their reliability and accuracy in classifying failure modes in CFRPs. The classification models exhibited up to 99% accuracy, as depicted by evaluation metrics. Further studies, using cross-correlation techniques, ascertained the presence of fiber break events occurring in the bundles as the thermoset CFRP composite approached failure. These findings highlight the significance of integrating machine learning into SHM for the early detection of real-time damage and effective monitoring of residual life in composite materials.

## 1. Introduction

CFRP composites have emerged as essential materials in aerospace, automotive, and structural engineering applications due to their high strength-to-weight ratio, corrosion resistance, and design flexibility. Due to their complex and anisotropic nature, CFRP composites exhibit a range of failure modes, such as matrix cracking, fiber breakage, and delamination, that often occur concurrently and evolve under applied loads. Accurately detecting and distinguishing these damage mechanisms is essential for effective SHM and for predicting the service life of composite components. SHM has evolved with significant relevance, ranging from damage detection to studying the integrity of structures [1,2]. In the context of SHM, AE refers to the detection and analysis of transient elastic waves generated within a material because of active deformation, mechanical loading, or damage events. These waves, referred to as AEs, are produced when energy is released due to internal changes such as cracking, plastic deformation, or structural instability. Owing to its passive monitoring nature, AE has been widely applied across various engineering fields—including manufacturing, civil, aerospace, and materials engineering—to investigate fracture and failure mechanisms in materials such as composites, metals, and concrete [3]. Recent research has focused on correlating AE signals to specific damage mechanisms using machine learning. Studies have demonstrated the effectiveness of various algorithms, such as k-nearest neighbors [4], support vector machines [5], and hierarchical Bayesian inference [6], in classifying AE signals and identifying damage types, such as matrix cracking, delamination, fiber breakages, and debonding. Correlations have also been established between damage mechanisms and specific AE signal features, including frequency bands and energy content [7]. Sai-nan et al. [8] proposed a method for damage signal recognition in carbon fiber composites using modal acoustic emission and machine learning with high accuracy in damage classification and location prediction, hence providing a reference for the SHM of composite materials. Dabetwar et al. [9] explored the use of signal processing techniques to improve machine learning algorithms for damage classification in composite materials by reducing the required number of features while improving their performance. Some studies explore the use of machine learning techniques for efficient damage classification of composite materials using features extracted from acousto-ultrasonic measurements, contributing to advanced online SHM strategies [10].

The damage mechanisms in composite materials are inherently complex and are significantly influenced by the interaction between the fiber and matrix components, the nature of the applied load, and the structural ply orientation itself [11]. Common failure modes such as matrix cracking, delamination, and fiber breakage can cause serious structural degradation under both quasi-static and fatigue conditions, often leading to catastrophic failure. Numerous studies have explored how these damage modes originate and evolve. For instance, Yokozeki et al. [12] reported that transverse matrix cracks consistently initiate at 90° interfaces in angled plies, with micro-cracks subsequently spreading into 0° plies. They also noted that the thickness and orientation of surrounding plies influence the extent and direction of crack propagation. In a related study, Kashtalyan and Soutis [13] examined matrix cracking in off-axis plies of unbalanced laminates and found that both normal stress perpendicular to the fibers and shear stress along the fiber direction play key roles in crack development. Mixed-mode fractures (combining Mode I and Mode II) were also observed due to shear-extension interactions in symmetric laminates.

Tessema et al. [14] found that failure in quasi-isotropic composite materials started out as a matrix crack and gradually propagated to the nearby plies and eventually grew to a delamination. Masters et al. [15] investigated cumulative damage in graphite/epoxy laminates under quasi-static tension and tension–tension fatigue. They used two types of laminates ([0/±45/90]_s_ and [0/90/±45]_s_) to compare the evolution of matrix crack damage. Negligible damage was observed in the outermost 45° ply for ([0/±45/90]_s_) laminate when the saturation spacing was computed, while matrix cracks were also present for the other orientation in the off-axis plies. Travis et al. [16] performed a study on biaxial fatigue damage mechanisms in quasi-isotropic CFRP composite laminates, focusing on early-stage damage and the physics of failure under complex loading using advanced techniques to analyze damage propagation and stiffness degradation. Specimen stiffness was measured and correlated with micro- and macroscale damage mechanisms and loading parameters. The results provided insight into the initiation and propagation of damage mechanisms in these materials. Ujjin et al. [17] monitored damage development and progression during pin loading of a hole in a quasi-isotropic carbon fiber reinforced composite and found that the sequence of damage evolution changes with the type of material discontinuity present. The analysis showed that failure was initiated by fiber matrix debonding, followed by fiber fracture and subsequent matrix cracking. They also confirmed that AE monitoring combined with FFT frequency analysis is an effective way to detect damage initiation and monitor subsequent micro failure events.

Under monotonic tensile loading, the density of matrix cracks and delaminations tends to be lower than in specimens subjected to fatigue. Observations revealed clusters of adjacent fiber breaks, with damage often spreading into the 0° plies in the form of isolated fiber failures, particularly near matrix cracks in off-axis plies. Beaumont et al. [18] supported this finding, emphasizing that the progression from matrix crack to delamination or fiber failure is governed by localized stress conditions.

Pradhan et al. [19] used finite element models to analyze the effect of transverse cracks in 90° plies on stiffness degradation in angle-ply laminates. They found that laminates with a greater number of ±45° outer plies exhibited only a modest reduction in stiffness and that increased ply count significantly improved resistance to crack propagation, highlighting the structural role of outer layers in constraining damage growth.

Kumar et al. [20] investigated the progression of fiber breakage in aerospace-grade unidirectional composites under tension. Using fractography, they observed unstable fracture patterns linked to the simultaneous failure of neighboring fibers. Their findings also suggest that strong interfacial bonding can promote crack propagation from fiber breaks into the matrix. Scott et al. [21] employed computed tomography to examine failure progression, such as matrix cracks, delaminations, and longitudinal splits, in double-edge notched cross-ply laminates. At lower loads, isolated fiber breaks and small clusters were observed, while higher loads caused larger clusters to emerge within a narrow stress range, rather than gradually increasing with load. Accurately identifying and quantifying AE signals related to different failure mechanisms in composite materials is crucial for evaluating structural damage progression and estimating the remaining safety margin before catastrophic failure. To address this need, researchers have proposed various AE signal characterization approaches tailored to different failure modes. These include classifications based on individual parameters, such as amplitude or frequency, multi-parameter pattern recognition techniques, and analysis focusing on extensional and flexural wave modes, commonly referred to as modal AE [22]. While amplitude-based classification has been widely investigated, its effectiveness in distinguishing between failure modes has proven inconsistent, as demonstrated by prior studies [23,24]. Similarly, methods using peak frequency content have shown some reliability [22,25], although their applicability is often constrained by the frequency response of the AE sensors used.

More recent efforts have explored machine learning—both supervised and unsupervised—to classify AE signals using traditional waveform-derived features [26]. However, these methods are sensitive to acquisition settings, which can affect the consistency and accuracy of the features used for classification. In contrast, modal AE techniques [27] have shown promise in improving classification accuracy. Although single-parameter methods may lack robustness, Baker et al. [28] demonstrated that integrating modal AE analysis with peak frequency evaluation effectively identifies transverse matrix cracks in the 90° plies of quasi-isotropic and cross-ply laminates. Recent studies have explored the use of AE together with machine learning techniques for monitoring damage in metals and composite materials. The results from these analyses have shown promising results in predicting and classifying the various failure modes [29,30]. Muir et al. [31] offer a comprehensive overview of machine learning models used for damage detection in composites, with particular attention to waveform-based feature extraction techniques. Nasiri et al. [32] used supervised learning methods such as convolutional neural networks and random forests to predict damage stages in SiC_f_-SiC_m_ composites with high accuracy. Almeida et al. [4], using the k-nearest neighbor algorithm, achieved 88% accuracy in classifying AE signals from different damage mechanisms in commercial composite structures. Other studies have shown that machine learning models can effectively predict fatigue life and characterize damage progression in various composite materials, including glass fiber-reinforced polyester and carbon fiber-reinforced polymer composites [10,33,34,35].

To effectively analyze and quantify bundles of fiber breaks in thermoset CFRPs, this study utilizes cross-correlation techniques. Cross-correlation, a key signal processing method, is widely applied in the analysis of AE signals to identify localized damage and track its progression by assessing the number and size of damage clusters. In the context of unsupervised learning, cross-correlation has proven effective for clustering fiber-break signals based on their amplitude and frequency characteristics [36,37]. Additionally, Rijal et al. [38] successfully used cross-correlation to classify different failure modes in both thermoset and thermoplastic composites, using visually labeled data. This technique also enabled the analysis of fiber break clusters and the evolution of AE energy through the resulting clusters.

In this study, machine learning models and techniques are used to characterize the three main failure modes in quasi-isotropic CFRP composites, that is, Matrix Cracking, Fiber Breaks, and Delamination. Different feature extraction and feature selection techniques were explored to acquire the best combination of features for training the machine learning models.

## 2. Background

### 2.1. Acoustic Emission System

**Figure 1:**
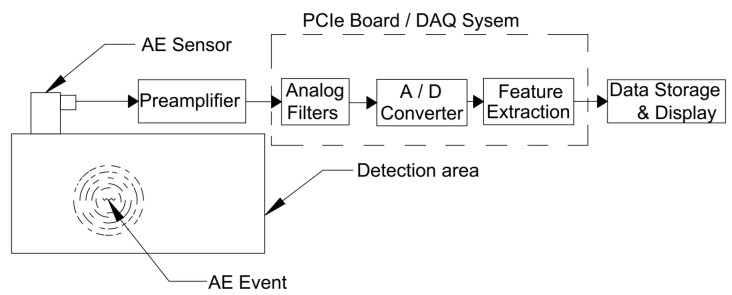
Schematic for AE acquisition system.

The AE testing system primarily relies on sensors mounted on the surface under inspection. These sensors convert mechanical energy in the form of vibration, stress, or axial strains into an electric voltage [39], which is then processed by a data acquisition system that includes preamplifiers, analog filters, analog-to-digital converters, and storage units, as seen in Figure 1. The characteristics of the AE signals captured through this system are primarily influenced by the source of the emission, the propagation medium, and the quality of both the sensors and the associated electronic equipment.

**Figure 2:**
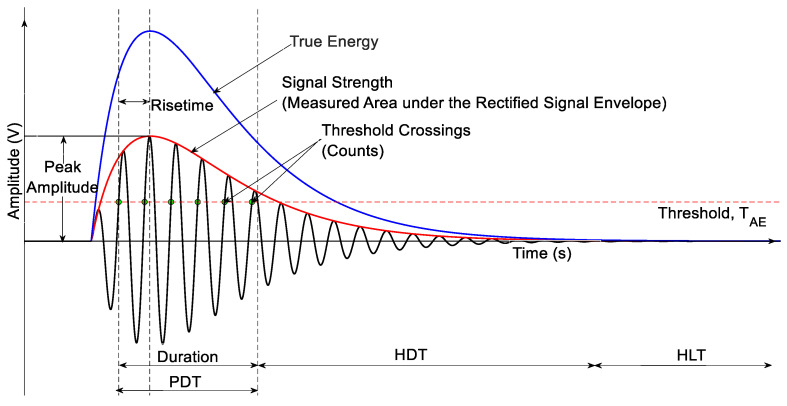
Schematic for AE Parameters and acquisition parameters.

AE signals are typically analyzed using either a parameter-based or signal-based approach [40]. In parameter-based analysis, specific features, referred to as AE parameters, are extracted from the signals. The calculation of these parameters is highly influenced by both the acquisition settings and the sensor characteristics used. Various AE signal parameters and acquisition parameters play a critical role in shaping these extracted AE features, some of which are shown in Figure 2.

#### 2.1.1. Conventional AE Parameters

The primary acquisition parameters applied before detecting and acquiring AE signals are discussed below:

1. Threshold: Refers to the minimum amplitude level (usually expressed in decibels, dB) that an acoustic signal must exceed to be recognized and recorded by the AE system.
2. Peak detection time (PDT): Defines the time window after the initial threshold crossing within which the system detects the peak amplitude of the AE hit.
3. Hit definition time (HDT): Defines the minimum duration that the AE signal must remain below the threshold before the system considers the current AE hit to have ended.
4. Hit lock time (HLT): Defines the fixed period after the end of a hit during which no new hits will be recognized, even if the signal exceeds the threshold again.

Similarly, some of the time domain conventional AE parameters that the data acquisition system calculates are as follows:

1. Hit start: Sample point before the first point over threshold (V ≥ T_AE_).
2. Hit end: Sample point after the last point over the threshold. (V < T_AE_).
3. Duration: The time between the hit start and the hit end sample points.
4. Counts: Refer to the number of times the AE signal crosses the set threshold level during a single AE hit.
5. Amplitude: It is the maximum amplitude of the signal, detected within the PDT at the sensor, converted to dB, given by

(1) $$Amplitude=20\;{log}_{10}\;\left(\frac{V_{max}}{V_{ref}}\right)-preamplifier\;gain$$

where V_max_ is the max signal amplitude in V, V_ref_ = 1 μV, and pre-amp-gain is in dB.

6. Rise time: Defines the time from the hit start to the max amplitude sample point.
7. Counts to peak: Number of threshold crossings that occur between hit start and peak amplitude.
8. Signal strength: Defines the area under the rectified signal. It is given by the measured area under the rectified signal envelope (MARSE).
9. Absolute energy: Defines the true energy of the signal on a 10 k Ohm resistor computed at the sensor.

Signal-based AE analysis uses the actual AE waveform recorded during the test to interpret the fundamental mechanisms. Although signal-based AE analysis provides greater reliability and a deeper understanding of the underlying physics of the AE source, it is generally more costly and time-consuming than parameter-based analysis due to the need for extensive data processing and a dedicated post-processing environment [41]. However, recent advances in signal processing, data management, and computational speed have significantly enhanced the efficiency and practicality of signal-based AE analysis, allowing for a more accurate interpretation of AE sources.

#### 2.1.2. AE Waveforms and Failure Events in Composites

The recorded AE waveform can be represented using a transfer function [42] as

(2) $$H_{AE}=H_{S}\times H_{M}\times H_{T}\times H_{E}$$

H_S_, H_M_, H_T,_ and H_E_ are the transfer functions for the source, acoustic wave propagating media, sensor properties, and electronics involved, respectively. The acoustic emissivity of a material, which quantifies its efficiency in converting stored strain energy into detectable acoustic waves, is contingent upon both the material’s intrinsic properties and the failure mechanism. Consequently, brittle materials, characterized by rapid, localized fracture, exhibit a higher acoustic emissivity compared to ductile materials, where energy is dissipated over a larger volume through plastic deformation [43]. Furthermore, due to the inherent brittleness of thermoset-based CFRP [44], high-fidelity AE signals are generated during damage events, which can be effectively captured by AE sensors.

The underlying mechanisms of AE source events are anticipated to generate distinct AE signals, each characterized by unique waveform features. The failure modes investigated in this study, matrix cracking, delamination, and fiber breaks, therefore produce AE signals with distinguishable signal characteristics reflective of their respective damage mechanisms. The fiber-break events, which are shorter in duration, are seen to produce high-frequency AE signals with a duration greater than 30 µsec [45]. On the other hand, matrix crack failure modes typically produce signals with durations < 100 µsecs and delamination events with medium-to-long durations (>120 µsecs) [46]. Other studies by Malolan et al. [47] showed that delamination events generated medium to high amplitude signals with longer durations (>100–200 µsecs). Rijal et al. [48] modeled the failure modes in composite materials using numerical analysis and developed frequency ranges of damage mechanisms for fiber breaks, matrix cracking, and delamination. The frequency ranges were cross-correlated with actual experimental data and showed a good correlation, establishing the basis for the frequency ranges used in this study. They also proved that delamination events produced mainly low-frequency signals (<250 kHz), matrix crack events having frequency content of up to 650 kHz, whilst fiber breaks with frequencies extending over 2 Megahertz (MHz). Furthermore, they also used modal acoustic analysis to characterize the AE waveforms obtained from these failure mechanisms based on the presence of Lamb wave modes. Delaminations were observed to have a dominant low-frequency fundamental antisymmetric mode, matrix cracks were seen to have fundamental symmetric and antisymmetric modes with varying amplitudes based on the source location about the neutral axis, with fiber breaks comprising higher-order modes. Moreover, the attenuative behavior of CFRP laminates introduces a frequency-dependent damping phenomenon, wherein high-frequency AE signals undergo more pronounced energy dissipation compared to lower-frequency signals [49]. This phenomenon results in pronounced attenuation of low-amplitude, high-frequency AE events, such as fiber breakage, whereas AE signals associated with delamination, characterized by higher amplitude and lower frequency content, exhibit greater propagation distances. Consequently, a distributed sensor array is required to ensure accurate detection and localization of these varying AE signals.

### 2.2. Machine Learning Algorithms

To predict the failure mode in the tested specimens, multiple machine learning (ML) algorithms were implemented and evaluated for comparison. These algorithms were chosen due to their proven effectiveness in classification objectives and their varied approaches to modeling relationships in data. The machine learning algorithms used are highlighted below.

Logistic Regression (LR) was considered due to its wide application for multiclass classification tasks. It uses regularized logistic functions to model the relationship between the input attributes and the output class. This research implements L2 regularization as a penalty parameter to keep the coefficients relatively small, while preventing zero-value coefficients [50]. The comparative study on classification performance by Musa [51] demonstrates LR’s efficiency and interpretability of results. The k-Nearest Neighbors (KNN) algorithm, also considered for this research, works by finding the k-nearest data points (neighbors) from a training dataset to a given query, based on the closest distances. After identifying these k neighbors, a majority-voting rule is applied to determine the final classification for the query [52]. The ability of KNN to capture patterns increases its effectiveness for even complex datasets [53]. In this research, the KNN algorithm is set to use specific training parameters such as five (5) neighbors, uniform weights, and a thirty (30) leaf size for classifying the failure modes. Linear Discriminant Analysis (LDA), as a dimensionality reduction and classification technique used in this research, projects the data onto a lower-dimensional space where class separability is maximized. It is capable of finding a linear combination of features that characterizes multiple classes [54,55]. LDA specifically requires the independent variables to be continuous and the dependent variables to be categorical, which are satisfied by the training data used for this research. Liu et al. [56] proved the excellent performance of LDA in reducing redundant features in multidimensional characteristic parameters for efficient classification.

Ensemble learning algorithms leverage diversity among several individual models by combining multiple models (weak learners) to improve the overall prediction accuracy. The superiority of the ensemble learning algorithm was demonstrated by the Random Forest classifier in classifying damage levels in CFRP composite materials [53]. The Random Forest (RF) builds multiple decision trees during training and averages their predictions to enhance accuracy and generalizability. The RF approach is implemented in this research by specifying a hundred (100) estimators (Decision Trees), enabling the model to better capture patterns by reducing variance. The Decision Tree classifier splits the data based on feature thresholds to create a tree-like structure for decision-making. The minimum number of samples for a leaf (“min_samples_leaf”) and the minimum number of samples to split an internal node (“min_samples_split”) were specified to be one (1) and two (2), respectively. No maximum depth (“max_depth”) was defined for the trees to allow the nodes to expand until all leaves contained fewer than the “min_samples_split” samples. Milad et al. [57] obtained the best prediction accuracy for the RF model in designing a fiber-reinforced polymer composite strain prediction model from multiple ensemble learning algorithms. These algorithms are also considered due to their ability to interpret and adapt to any nonlinear relationships in the dataset used for this research.

### 2.3. Performance Evaluation

The performance of the machine learning models needs to be effectively evaluated using appropriate metrics. This enables a successful comparison of the models. This section highlights the evaluation metrics used in this paper and how they can be estimated.

#### 2.3.1. Cross-Validation

Cross-validation (CV) is a model evaluation method in which a dataset is resampled and divided into two portions: one is used to train a model, and the other is used to validate the model. In this paper, a Repeated Stratified K-fold CV is implemented. This method, first, performs a k-fold CV [58] ten (10) times. The process was set to be repeated three (3) times with different random data splits. The stratification ensures that the output (class) distribution is maintained in each fold. The Repeated Stratified K-fold CV enhances a robust estimation of the model performance by reducing variability in scores due to different splits [58].

#### 2.3.2. Confusion Matrix

The confusion matrix is a comprehensive tabular presentation of a model’s performance. It shows a clear view of both the success and failure of a model using the estimated true positive (TP), false positive (FP), true negative (TN), and false negative (FN) [59].

#### 2.3.3. Precision

Precision measures the correctly predicted positive cases (TP) compared to the total predicted positive cases (TP and FP). High precision implies that a prediction will likely be correct if the model predicts a positive class. It is crucial in instances where false positives can have a significant impact.

(3) $$Precision=\frac{TP}{TP+FP}$$

#### 2.3.4. Recall

Recall, also known as sensitivity, measures a model’s tendency to identify the positive cases from all the actual positive cases available. A high recall shows how successfully a model can identify most of the positive instances in the dataset.

(4) $$Recall=\frac{TP}{TP+FN}$$

#### 2.3.5. Accuracy

Accuracy measures the correct predictions out of all predictions made by the model. It considers both positive and negative predictions.

(5) $$Accuracy=\frac{TP+TN}{TP+TN+FP+FN}$$

#### 2.3.6. F1 Score

The F1 score provides the harmonic average of precision and recall. It gives a balanced estimate of a model’s accuracy when the dataset is uneven, while recognizing the importance of precision and recall.

(6) $$F1\;Score=2\times \frac{Recall\times Precision}{Recall+Precision}$$

The combination of the evaluation metrics obtained from each trained model is used to quantify or weigh the validity of each model’s predicted class. Aside from identifying the most appropriate ML algorithms for the classification task, the final class can be voted on by assigning greater weights to models with higher evaluation metric values.

### 2.4. Cross-Correlation

AE signals recorded during experiments are influenced by four primary factors, as given by Equation (1). If the effects of sensors and electronic components remain consistent throughout the experiments, achieved with high-fidelity sensors and reliable data acquisition equipment, the acquired AE signals can be considered primarily dependent on the source characteristics and the propagation path. Under these conditions, cross-correlation, which quantifies the similarity between two signals, becomes a valuable tool for identifying AE signals originating from similar sources and locations. Eaton et al. [60], when studying the similarity of AE signals in CFRP composite panels, found that signals with the same source-to-sensor distance had a cross-correlation coefficient greater than 0.90. For a discrete time series signal, x(t) and y(t), the normalized cross-correlation coefficient for shifted copies of y(t) at τ, called the lag, is given by:

(7) $$\rho_{xy}\left(\tau \right)=\frac{\sum_{i=0}^{N-1}\left(x_{i}-\bar{x}\right)\times \left(y_{i-\tau }-\bar{y}\right)}{\sqrt{\sum_{i=0}^{N-1}{\left(x_{i}-\bar{x}\right)}^{2}}\times \sqrt{\sum_{i=0}^{N-1}{\left(y_{i-\tau }-\bar{y}\right)}^{2}}}$$

where $\bar{x}and\bar{y}$ are mean of x(t) and y(t) and τ is the number of data points to shift signal y(t).

## 3. Materials and Methods

### 3.1. Experimental Procedures

**Figure 3:**
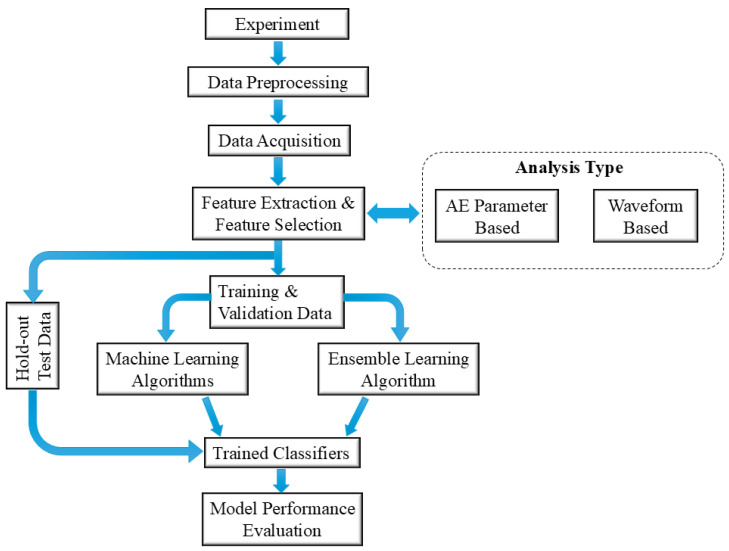
Proposed Classification Framework.

Figure 3 shows the proposed framework for classifying the failure modes. A quasi-isotropic thermoset CFRP coupon with lay-up sequence [45/90/−45/0]_2s_, was subjected to quasi-static tensile loading until failure. AE signals from the different failure modes were obtained and processed during the experiment using bonded Lead Zirconate Titanate (PZT) sensors. The signals were further processed to extract features for training the machine learning models. The detailed procedures are as follows.

#### 3.1.1. Sample Preparation

The test specimens were prepared according to ASTM standard D3039 [61]. Thermoset carbon fiber-reinforced epoxy quasi-isotropic laminates with lay-up sequences [45/90/−45/0]_2s_ having nominal dimensions 12″ × 1″ × 0.094″ were used for the tensile test. The composite panel was made up of IM6/3501-6 unidirectional prepreg cured in an autoclave at 85 psi pressure and a temperature of 240 °F. Glass-epoxy tabs were attached to the ends of the specimen to enable secure gripping. The tabs were tapered at a 10° angle to reduce stress concentrations at the interface.

#### 3.1.2. Instrumentation

**Figure 4:**
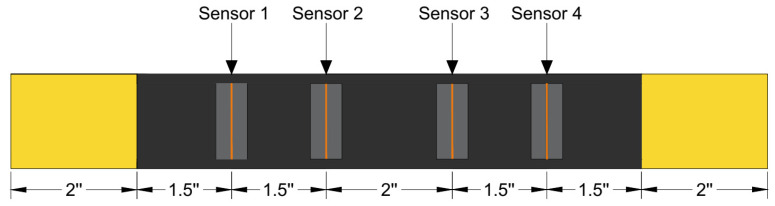
Figure illustrating the position of the sensors on the composite coupon.

Y. Bhuiyan et al. [62] showed that the type of AE sensor used has a significant effect on the captured AE waveform and its corresponding frequency spectrum. Piezoelectric wafer active sensors (PWAS) showed a better signal-to-noise ratio in the high-frequency region than commercial AE sensors. PWAS can capture both axial and flexural modes of lamb waves, making them well-suited for studying AE in thin, plate-like structures [62]. Consequently, custom-fabricated bonded PZT sensors with a frequency response of up to 3 MHz [63] were used in this study. PZT-5A wafers of dimension 15 mm × 7 mm × 0.2 mm and a sensing aperture of 1 mm with steel electrodes were bonded to the surface of the specimen to acquire AE signals by strategically placing the sensors at well-determined locations, as illustrated in Figure 4.

#### 3.1.3. Loading

The testing specimens were subjected to tensile loading using an MTS 810 material testing system until failure. The specimen was loaded using a loading rate of 300 lbf/min (1.3345 kN/min), and the AE data were acquired for further processing.

### 3.2. Signal Preprocessing and Data Acquisition

With a preamplifier gain of 60 dB, which is compensated during feature extraction, and a sampling frequency of 20 MHz, the PCI-2 AE data acquisition system from Physical Acoustics was used to record the AE waveforms. Designed for low-noise performance, the PCI-2 offers 18-bit A/D conversion with a maximum voltage of ±10 V. Low-amplitude noise signals were eliminated by setting a threshold of 40 dB and using a bandpass analog filter with frequencies ranging from 1 kHz to 3 MHz. Numerous waveforms were recorded during the experiment. AEwin (vE5.90) and Noesis (v12.0) software from MISTRAS Group were used for data acquisition and data visualization, along with post-processing, respectively.

The application of the threshold depends on various sources of noise. While mechanical noise can influence data acquisition, the dominant source of noise in bonded PWAS is electronic noise, primarily originating from the circuitry. The threshold is typically chosen so that no signals are detected under unloaded conditions. However, if the threshold is set too high, it can lead to inaccurate quantification of AE signals. For instance, a high threshold may cause low-amplitude, low-frequency symmetric modes to be missed, resulting in delayed triggering and misinterpretation of signal duration. Similarly, it may cause the later-arriving low-frequency, low-amplitude antisymmetric modes to be overlooked, leading to premature signal termination. The threshold of 40 dB was chosen to filter out signals unrelated to AE, since no AE activity is expected under unloaded conditions. Although using a lower threshold could capture signals not associated with AE from composite failure, the AE signals themselves would still be recorded at any threshold, as their amplitudes generally exceed all thresholds used in this study.

**Figure 5:**
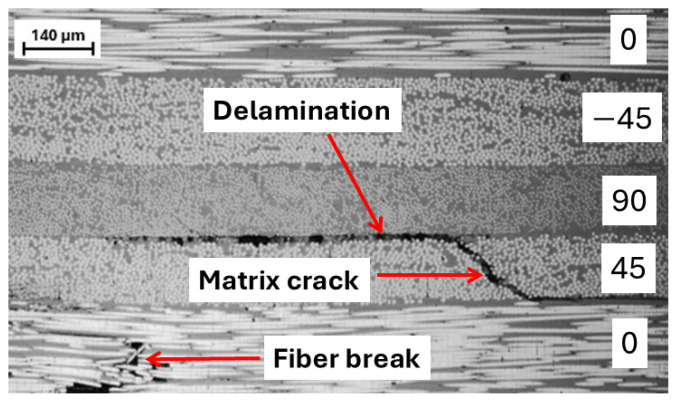
Micrograph illustrating the failure modes in composite materials.

Figure 5 shows the optical micrograph of the cross-sectional area of the composite laminate with the three failure modes. The micrograph was taken using ZEISS Axio Zoom.V16 under a magnification of 60x. In the 45° ply, the matrix crack can be noticed to advance into delamination between the 90° and 45° plies. The presence of fiber breaks can be seen in the 0° ply.

**Figure 6:**
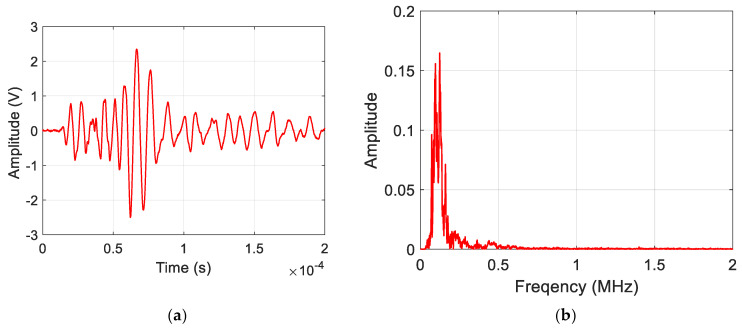
Sample (**a**) Waveform and (**b**) Frequency spectrum for Delamination damage mode.

**Figure 7:**
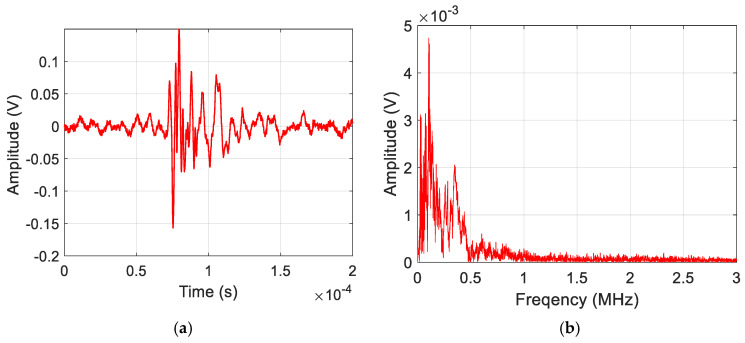
Sample (**a**) Waveform and (**b**) Frequency spectrum for Matrix Crack damage mode.

**Figure 8:**
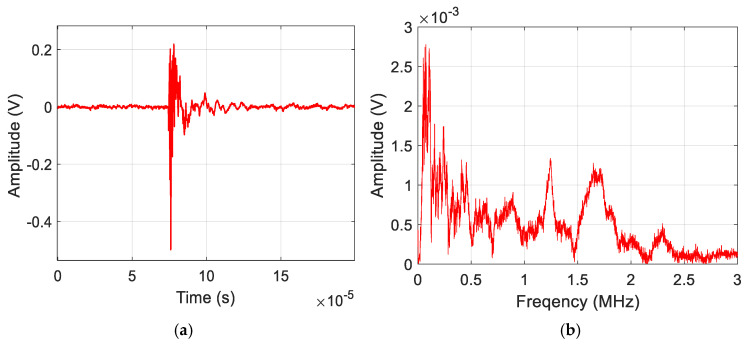
Sample (**a**) Waveform and (**b**) Frequency spectrum for Fiber Break damage mode.

Figure 6 shows a representative AE signal of a delamination event acquired during the experimental analysis. The waveform is characterized by high amplitude and very low-frequency content (<225 kHz). Figure 7 shows an AE signal obtained from a matrix crack event. They possess frequency content ranging to 650 kHz. Figure 8 shows an AE signal for fiber break events, and they are characterized as having the highest frequency content, ranging to 3 MHz. The representative waveforms are samples of amplified AE signals of the failure modes within the specified frequency ranges.

### 3.3. Feature Extraction

To ensure the extraction of useful and informative features for machine learning models, both parameter-based and waveform-based AE analyses are utilized in this study. Parameter-based analysis provides statistical descriptors such as amplitude, duration, and energy, while waveform-based analysis captures detailed signal characteristics through processing techniques like filtering and transformation.

#### 3.3.1. Parameters-Based Feature Extraction

As discussed in Section 2.1, the acquisition parameters are seen to highly influence the traditional AE parameters. Hence, different acquisition settings were applied to extract common AE parameters, as shown in Table 1, readily available from the acquisition software. Conventional AE features were extracted using multiple thresholds and hit definition time settings during data post-processing to evaluate the influence of acquisition settings on model performance. The respective acquisition parameters used to create multiple datasets are shown in Table 2.

#### 3.3.2. Waveform-Based Feature Extraction

Waveform-based feature extraction was implemented by post-filtering the recorded waveforms during the experiment. Three bandpass filters were designed to correspond with the frequency characteristics previously identified for each of the three failure modes. The three frequency bands are given in Table 3.

The maximum amplitude of waveforms filtered using all three band-pass filters was then normalized using the amplitude of the original waveforms. This amplitude ratio indicates how much each frequency band contributes to the overall amplitude, and consequently, to the energy of the original signal.

### 3.4. Feature Selection

**Figure 9:**
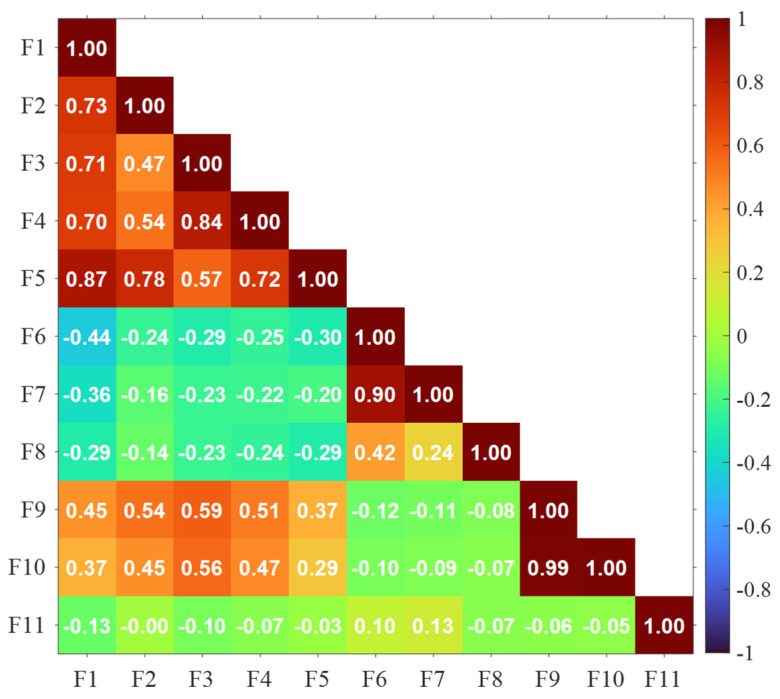
Correlation matrix of all features as seen in Table 1.

Feature selection is a crucial step in building robust machine learning models, aimed at identifying and retaining the most relevant features while eliminating redundant or irrelevant ones. One common and effective method for feature selection is the multicollinearity test, which is an unsupervised feature selection method that involves analyzing the correlation matrix of the dataset [64,65]. The correlation matrix displays the pairwise correlation coefficients between features, where a high correlation suggests linear redundancy among them. Figure 9 shows the correlation matrix of features as listed in Table 1, obtained from Specimen 1. The entire dataset from the thermoset CFRP composite (specimen 1) was used for the feature selection. A strong linear correlation is observed between the feature pairs (F1, F5), (F3, F4), (F6, F7), and (F9, F10). Correlated features are then subsequently removed from the dataset to enhance the model’s interpretability by reducing redundancy among variables and to improve generalization by minimizing the risk of overfitting to training data. A similar trend in correlation was observed in class-wise feature correlation analysis.

**Figure 10:**
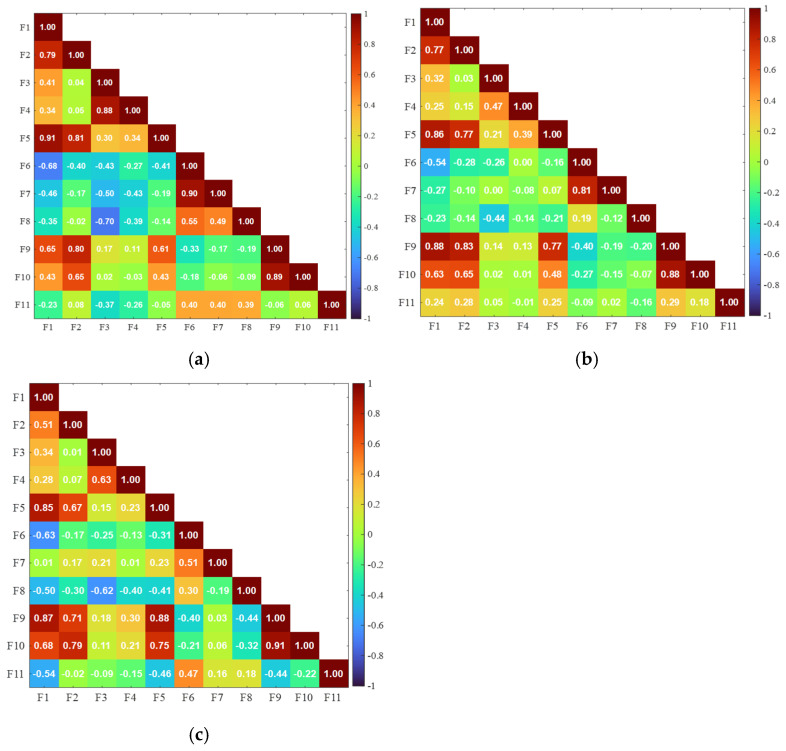
Correlation matrix of the features based on only (**a**) Delamination, (**b**) Fiber breaks, and (**c**) Matrix cracks.

Figure 10 presents the correlation matrix of features for the selected failure mode. The feature correlations identified through the unsupervised multicollinearity test are consistently observed across most failure modes, with no additional correlations emerging within the feature set. Therefore, the correlations detected in the unsupervised multicollinearity analysis are representative and inclusive across all evaluated failure modes.

**Figure 11:**
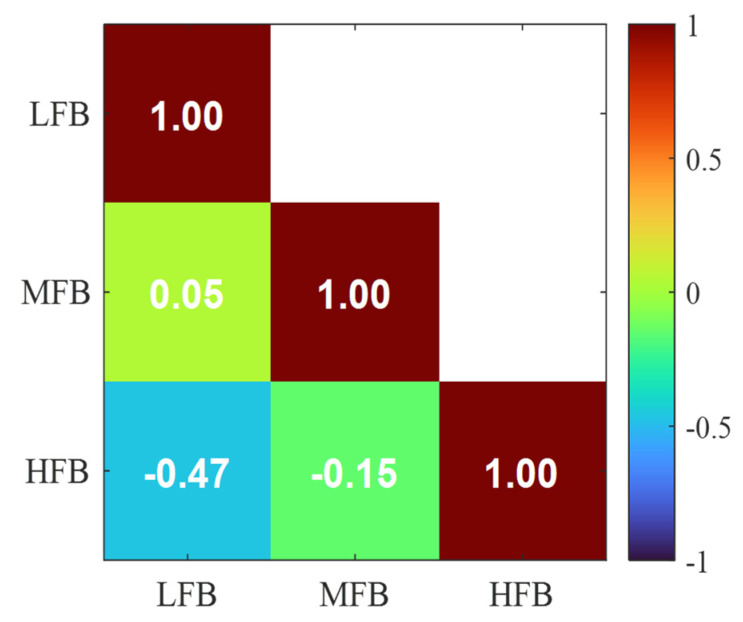
Correlation matrix for amplitude ratios extracted from the waveform.

Similarly, Figure 11 shows the correlation matrix for the amplitude ratios extracted from the filtered waveforms using the previously defined frequency bands. The absence of strong linear relationships among the features indicates that they are largely independent, making them well-suited for classification tasks.

Finally, the class labels were converted into numerical forms using the LabelEncoder class from the scikit-learn library. It enhances the performance of the classification models because many ML algorithms work better with numerical data than text data. Label Encoding is implemented over other encodings due to its ability to avoid an increase in the dimension of the data [66].

Before training the models, the dataset was split into a 60:20:20 ratio, with 60% of the data allocated for training, 20% for validation, and the remaining 20% for a hold-out test. The hold-out test is conducted to assess the performance and generalizability of the classification models on unseen data.

## 4. Results and Discussion

MATLAB R2024a and Google Colab Python 3.9 were used for preprocessing the training data and training the model, respectively, to meet computational demands. Google Colab offers a robust cloud-based environment with convenient computational resources for efficient analysis.

The features selected in Section 3.4 were used to train the ML models, discussed in Section 2.2, on a balanced dataset of seven hundred and fifty (750) AE signals from specimen 1. The dataset was prepared by manual inspection of the AE waveforms, considering their signal characteristics [48], which can also be seen in Figure 6, Figure 7 and Figure 8. For each damage class, 60% (150 sets of amplitude ratios) of the data was used for training, 20% (50 sets of amplitude ratios) for validation, and the remaining 20% (50 sets of amplitude ratios) for a hold-out test. A combination of the training (60%) and validation (20%) sets was used to perform the Repeated Stratified K-fold CV test. In contrast, the hold-out set was used solely to calculate the remaining evaluation metrics, including accuracy, precision, recall, and F1 score. The classes were encoded to facilitate the training process. The results of the evaluation metrics are discussed in the following sections.

### 4.1. Classification Performance Using Conventional AE Parameters

**Figure 12:**
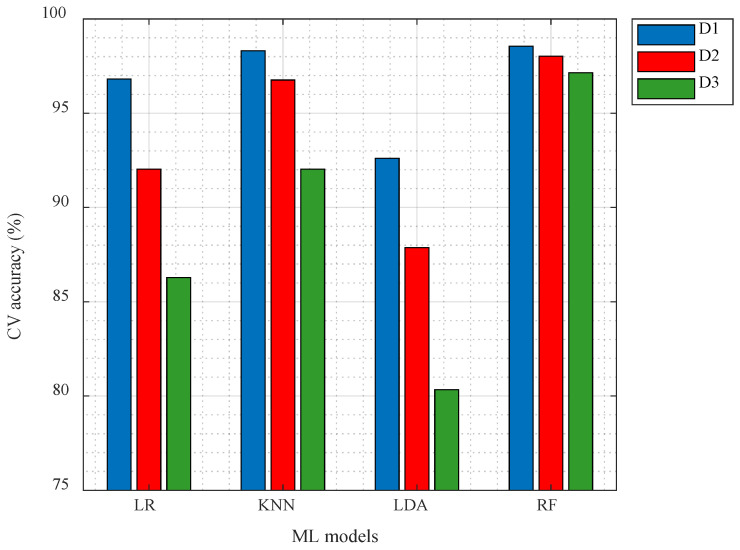
Variation in cross-validation accuracies for different ML models.

A total of seven parameters, as shown in Table 4, identified after conducting a multicollinearity test, were used as features to train four machine learning models employing the LR, KNN, LDA, and RF algorithms. Similarly, three different datasets were created by changing the acquisition parameters during feature extraction. Figure 12 shows the variation in cross-validation accuracies of the ML models for different datasets defined in Table 2. As seen from the figure, the accuracies in all models are seen to decrease across different datasets, clearly indicating the influence of acquisition parameters in AE features and hence the models’ performance.

The limitations of using the AE parameters in Table 4 to assess damage mechanisms in CRFP have been widely investigated by researchers. Godin et al. [26] demonstrated the unreliability of the AE signal parameters acquired from the data acquisition software in classifying different types of damaging events in a unidirectional glass/polyester composite. They implemented a post-processing analysis to develop new features for the classification using a floating threshold. Barile et al. [11] also presented a comprehensive review of the application of AE descriptors (parameters) in the damage assessment of fiber-reinforced plastics. The influence of the parameters on different types of damage evolution was studied, and it turns out that the peak frequency is one of the parameters that cannot be trusted as a solitary parameter for damage characterization.

### 4.2. Classification Performance Using Waveform Analysis

**Figure 13:**
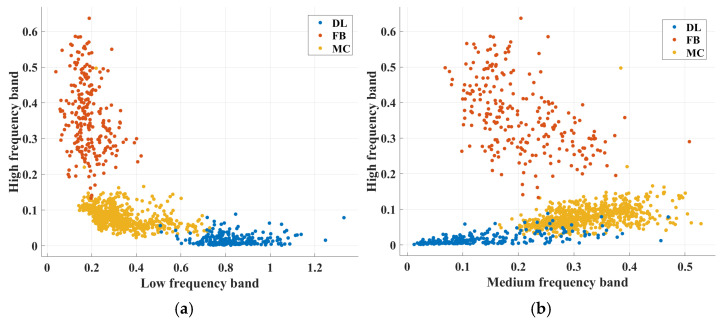
Distribution of amplitude ratios for (**a**) high-frequency band versus low-frequency band. (**b**) High-frequency band versus medium-frequency band.

Amplitude ratios obtained from three distinct band-pass filters applied to recorded AE signals were used as input features for developing machine learning (ML) models. Figure 13 shows the distribution of amplitude ratios across different frequency bands. The figure highlights the distinctions in amplitude ratios associated with the different failure modes under consideration. Fiber break (FB) events exhibit a significant energy contribution in the high-frequency band, whereas delamination (DL) events show dominant energy contributions in the low-frequency band. As previously discussed, matrix cracks (MC) demonstrate energy contributions from both the low- and medium-frequency bands.

**Figure 14:**
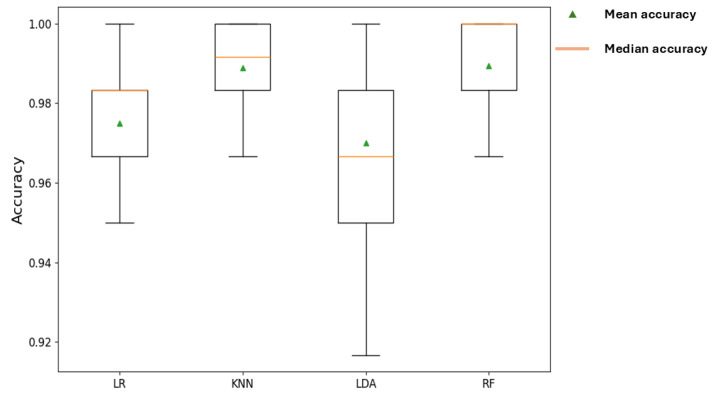
Model Performance Comparison.

Table 5 shows the accuracy scores of the Repeated Stratified k-fold CV of each classification model. The results indicate the outstanding performance of the ML models, with the KNN model exhibiting the highest accuracy of 98.89%. Figure 14 represents a boxplot of the accuracy scores for each model across all the folds of the repeated stratified k-fold CV. A boxplot is a statistical tool that summarizes the pattern or distribution of information in a dataset or results. It constitutes descriptive elements such as the Median, Interquartile Range (IQR), and Whiskers [67]. From the results, the consistently high accuracy scores, with medians for all models above 96% and top performers, such as KNN and RF, approaching 99%, show that the models have effectively learned the underlying patterns in the data. Furthermore, the stability of the top-performing models, KNN and RF, is an excellent indicator of good generalization. Their tight interquartile ranges (the small boxes) and short whiskers show that they performed consistently well across multiple different subsets of the data. However, while LDA yielded a decent mean accuracy, the very long whiskers, stretching from about 0.92 to 1.00, demonstrate a wide variance in its performance, making it the least reliable choice in accurately classifying the failure modes. Overall, this performance highlights the models’ ability to effectively analyze the patterns in the decision boundaries of the dataset. Thus, subsequent analysis will focus on the results obtained using the filtered AE signal features.

To evaluate the performance of the classification models using the remaining evaluation metrics, the trained models were tested on the hold-out set of the dataset. Table 6 presents the results of the machine learning models after training and testing on unseen data.

**Figure 15:**
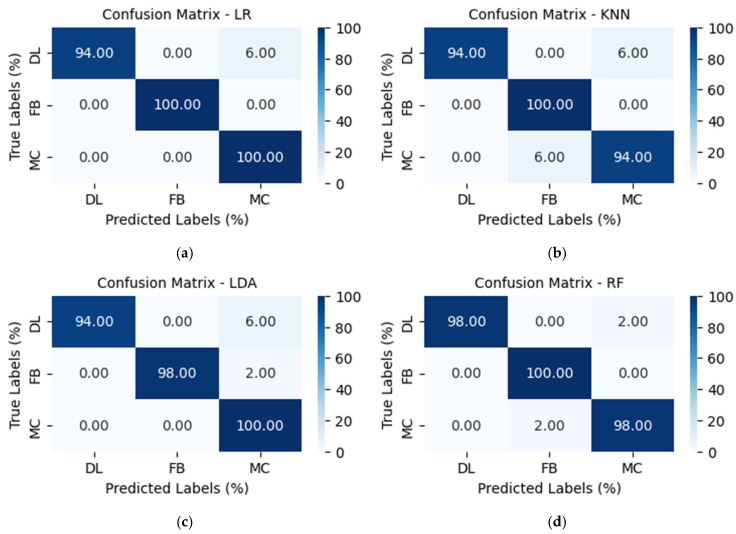
Confusion matrix for: (**a**) Logistic Regression. (**b**) k-Nearest Neighbor. (**c**) Linear Discriminant Analysis. (**d**) Random Forest.

The LR, KNN, and LDA models exhibited promising performance with accuracies of 98%, 96%, and 97%, respectively. They achieved near-perfect scores for the precision, recall, and F1-score for the failure modes. The imperfection in the performance is due to misclassifications among the failure modes, as shown in Figure 15a–c. For instance, they all misclassified 6% of delamination events as matrix cracks. The misclassification exhibited by the models suggests a potential imbalance in the models’ ability to capture the difference in failure modes, and calls for rigorous error analysis to identify any possible bias in the dataset.

The ensemble model, RF, demonstrated an almost-flawless performance in classifying the failure modes, achieving an outstanding accuracy of 99%, as shown in Table 6. Its ability to effectively study and reduce the complexity of the decision boundaries contributed significantly to the model’s performance. Out of the fifty (50) amplitude ratios for each damage class tested, the RF misclassified a delamination event as a matrix crack and a matrix crack event as a fiber break, as depicted by the confusion matrix in Figure 15d. Although the LR, KNN, and LDA classifiers had a minor setback in performance, the performance of the RF classifier dominated the final predictions of the failure mechanism.

### 4.3. Model Implementation

After successfully training and evaluating the classification models, the models were saved using the Joblib library. This tool enables model deployment and application on a new set of AE signal data. The models were used to perform further analysis on AE signals acquired from two different quasi-isotropic thermoset CFRP composite specimens. The remaining unlabeled AE signals from specimen 1, together with the entire test result from specimen 2, were used for further analysis. These specimens were made of the same material and tested under similar conditions. More than eight thousand (8000) AE signals acquired from each experiment using PZT sensors were preprocessed for classification. To ensure the highest confidence level, models with an accuracy of 100% were loaded to classify the failure mechanisms of the acquired AE signals. To identify and characterize bundles of fiber breaks in the thermoset CFRP, Random Forest’s probabilistic output was used to isolate the AE signals classified as fiber breaks by considering a probability of certainty above 90%.

**Figure 16:**
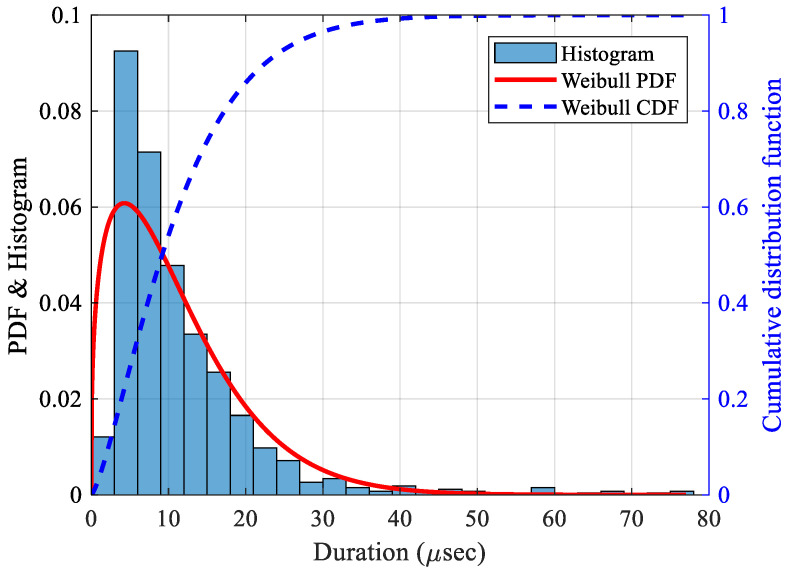
Distribution of duration for fiber-break events.

The waveforms of the fiber break events were then cross-correlated with a correlation coefficient of 0.90. The length of the window used for cross-correlating different events significantly affects the clustering process. Since fiber-break events are typically short in duration, the corresponding AE waveforms are also expected to be brief. To select a suitable window, a distribution analysis of events classified as fiber-break was conducted. The duration feature is significantly influenced by the source-to-sensor distance, presence of dominant symmetric and anti-symmetric mode shapes, and acquisition parameters, as discussed in Section 2.1, resulting in a highly skewed distribution as seen from the histogram in Figure 16. A Weibull probability distribution function (PDF) was then fitted, as seen in Figure 16, as it provides the best fit for positively skewed data [68]. The equation for the Weibull distribution is given by:

(8) $$y=\lambda \beta x^{\beta -1}e^{-\lambda x^{\beta }}$$

The distribution has a scale parameter (λ) of 13.17, meaning 62.3% of events have a duration less than 13.17 µsec, and a shape parameter (β) of 1.14, meaning a larger value skews the distribution to the right. With a confidence interval of 95%, λ and β have lower and upper bounds of [12.38, 14.01] and [1.09, 1.20], respectively. Similarly, the mean and variance for the distribution were 12.55 and 120.41, respectively. The significant difference between the mean and variance is due to the high skewness of the data. The duration of fiber break events typically varies, although they remain within a relatively short range, based on mode content and attenuation. Fiber break signals with higher frequencies are highly attenuated and hence die out quickly [49]. A suitable cross-correlation window of 40 microseconds (µs) was then selected, since the probability of fiber-break durations being less than this value was approximately 99%, as observed from the cumulative distribution function (CDF), which also agrees with the duration of fiber-break events as discussed in Section 2.1.2.

**Figure 17:**
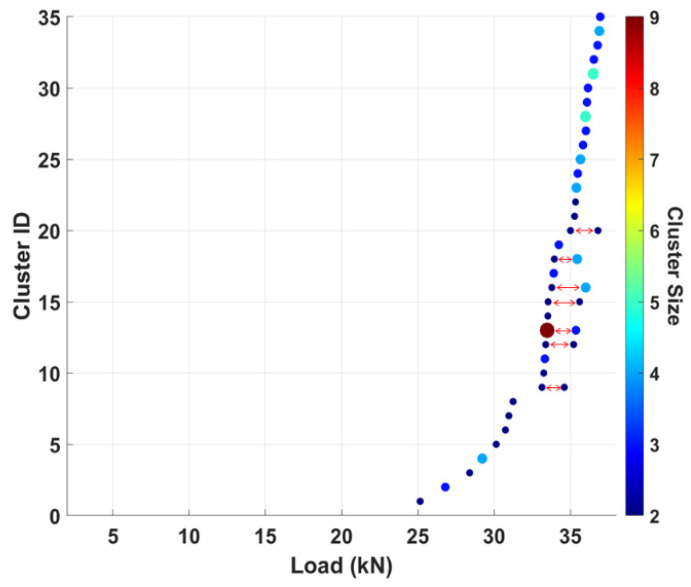
Specimen 1 plot showing clusters of fiber breaks for a 0.90 correlation coefficient.

The correlated signals are grouped into events occurring within the same loading rate to identify fiber break events that occurred as bundles. Figure 17 shows a plot of the clustered fiber break events against load for Specimen 1. The postprocessing result for specimen 1 revealed clusters of fiber break events with sizes ranging up to nine (9) as defined in Table 7. It can also be noticed that the number of clusters increases considerably as the specimen approaches failure.

The Cluster ID represents the number of unique AE waveforms that were correlated and grouped as clusters. For a correlation coefficient of 0.90, specimen 1 appeared to have a total of thirty-five (35) unique waveforms correlated. This implies that fiber breaks occurred in groups at 35 unique locations in specimen 1, considering a 90% degree of correlation. Similarly, a 90% degree of correlation yielded clusters of fiber break events from 28 unique locations in specimen 2, as indicated in Table 7.

In addition, multiple clusters of fiber break events were found to be highly correlated while occurring at different load levels, as indicated by the double-line arrows in Figure 17. Considering the signal characteristics, such as source-to-sensor distance, wave propagation path, frequency content, and sensor properties, that were incorporated in correlating the waveforms, it can be confirmed that the correlated multiple clusters occurred very close to each other. However, the difference in the load levels is due to the delay or impediment of fiber crack propagation by the viscoelastic nature of the matrix [69].

**Figure 18:**
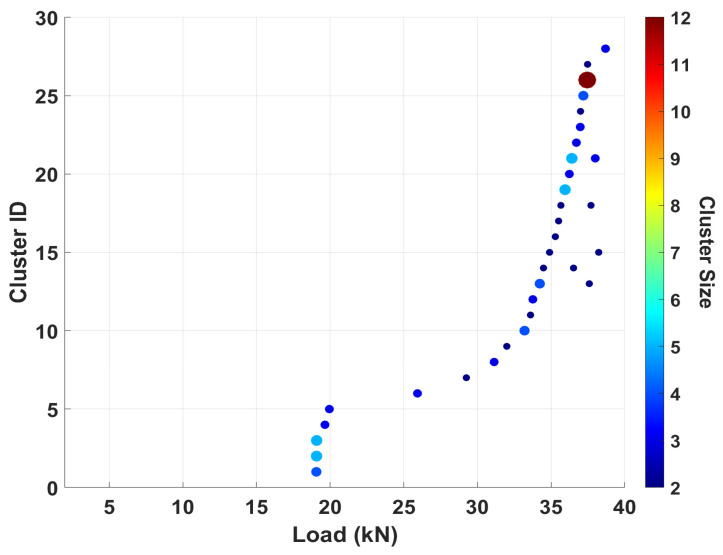
Specimen 2 plot showing clusters of fiber breaks for a 0.90 correlation coefficient.

Although Specimen 2 exhibits fewer distinct clusters compared to Specimen 1, the underlying failure mechanisms remain consistent across both specimens. As indicated in Table 7, Specimen 2 generated relatively larger cluster sizes. These discrepancies in cluster count and size of fiber break events can primarily be attributed to source and sensor location and the limited detectability of such events, which are typically characterized by short durations and low AE energy levels. In addition, Figure 18 shows a rapid cluster formation between 19 kN and 20 kN, followed by a lag in cluster formation until 34 kN. The clusters formed at an approximate load indicate the tendency of the carbon fibers to break in groups at different locations, but almost simultaneously. Additionally, the clusters of fiber breaks were also seen to increase drastically after the specimen had reached about 80% of its ultimate strength (~32 kN).

These classification models can be integrated into monitoring the residual strength of a thermoset CFRP composite in real time. A carefully designed data processing pipeline will increase the reliability of an automated system that classifies the real-time failure mechanisms of a thermoset composite to prevent catastrophic failure when it is in service.

One of the key limitations of this study is the use of pure, single-failure-mode signals for training the classification models. While this approach provides a controlled environment for understanding and detecting the distinct failure modes, it restricts the models’ ability to generalize to more complex, real-world scenarios. In practical applications, structural failures sometimes occur in the form of superimposed or mixed failure modes, where multiple failure mechanisms may occur simultaneously. This complexity could be addressed in further studies by implementing more advanced approaches such as deep learning. Additionally, the models developed in this study are constrained by their applicability to a specific set of material and structural parameters, such as ply orientation and thickness. The training dataset used was limited to thermoset CFRP composites with consistent ply orientation, thickness, and layup configuration. As a result, the trained models are not directly transferable to other material systems such as thermoplastic composites or those with different ply orientations, thicknesses, or stacking sequences. Future work aims to explore the relationships between damage mechanisms in thermoplastic and thermoset CFRP composites, as well as different ply orientations, using artificial intelligence (AI) tools such as deep learning.

## 5. Conclusions

This paper explores the application of machine learning algorithms, such as LR, KNN, LDA, and RF, to accurately classify the failure mechanisms in thermoset quasi-isotropic CFRPs, including fiber breaks, delamination, and matrix cracks. To achieve this, AE signals acquired from a quasi-static tensile loading experiment were thoroughly preprocessed to create a balanced dataset for training the classification models. The reliability of using the AE parameters as training features was investigated. The results from repeated stratified k-fold CV, coupled with inconsistencies in the AE parameters addressed by literature, affirmed that different AE system acquisition settings, such as threshold and HDT, significantly influence the recorded AE parameters. Thus, AE parameters cannot be used solely to classify failure mechanisms in CFRPs.

Furthermore, a feature extraction technique was implemented to improve the classification results. Amplitude ratios corresponding to the three main failure modes’ frequency bands were used to create a dataset for training and classification. Various machine learning techniques, including ensemble learning, were employed, with 60% of the data reserved for training, 20% reserved for validation, and 20% reserved for hold-out testing. The classification models yielded an incredible performance, with the RF classifier having the highest accuracy, and 99% accuracy was obtained after testing on the hold-out set. Using the dataset extracted from the amplitude ratios of the frequency bands, the classifiers effectively captured and modeled any complex pattern within the classes. The trained classification models were applied to comprehensive datasets of AE signals from two specimens subjected to a quasi-static tensile test to investigate the characteristics of fiber break events in a thermoset CFRP composite. Using cross-correlation for post-processing analysis, the results indicated the presence of an increasing number of fiber break events occurring in bundles as specimens approached failure.

Future work should address the limitations associated with the methodology implemented in this research by incorporating more advanced AI tools. Overall, this research establishes the foundation for an intricate approach to studying real-time failure mechanisms using machine learning and cross-correlation techniques.

## Figures and Tables

**Table 1:** Conventional AE features acquired from the data acquisition system.

Feature Name	Feature Number
Duration	F1
Amplitude	F2
Risetime	F3
Counts to Peak	F4
Counts	F5
Average Frequency	F6
Reverberation Frequency	F7
Initiation Frequency	F8
Signal Strength	F9
Absolute Energy	F10
Peak Frequency	F11

**Table 2:** AE acquisition settings used for feature extraction.

Dataset	AE Threshold (dB)	PDT (µsec)	HDT (µsec)	HLT (µsec)
D1	32	100	200	700
D2	35	100	300	600
D3	40	100	400	500

**Table 3:** Frequency bands used to filter AE signals.

Frequency Band	Frequency Range (kHz)
Low-frequency band (LFB)	25–225
Mid-frequency band (MFB)	250–650
High-frequency band (HFB)	700–3000

**Table 4:** Final AE parameters used for training ML models.

AE Parameters
Duration
Amplitude
Risetime
Average Frequency
Initiation Frequency
Absolute Energy
Peak Frequency

**Table 5:** Repeated Stratified k-fold CV results for filtered AE signal features.

Model	Accuracy
Logistic Regression (LR)	97.50%
k-Nearest Neighbor (KNN)	98.89%
Linear Discriminant Analysis (LDA)	97.00%
Random Forest (RF)	98.67%

**Table 6:** Summary of the classification models’ performance.

Model	Accuracy	Precision	Recall	F1-Score
LR	98%	DL: 100% FB: 100% MC: 94%	DL: 94% FB: 100% MC: 100%	DL: 97% FB: 100% MC: 97%
KNN	96%	DL: 100% FB: 94% MC: 94%	DL: 94% FB: 100% MC: 94%	DL: 97% FB: 97% MC: 94%
LDA	97%	DL: 100% FB: 100% MC: 93%	DL: 94% FB: 98% MC: 100%	DL: 97% FB: 99% MC: 96%
RF	99%	DL: 100% FB: 98% MC: 98%	DL: 98% FB: 100% MC: 98%	DL: 99% FB: 99% MC: 98%

**Table 7:** Clustered fiber break events.

Specimen	Unique Clusters	Total Clusters	Max. Cluster Size
1	35	42	9
2	28	33	12

## Data Availability

The original contributions presented in this study are included in the article. Further inquiries can be directed to the corresponding author.
